# Geographic Variation in the Bacterial Microbiota of *Rhipicephalus sanguineus* (Acari, Ixodidae) Across Environmentally Contrasting Regions of Mexico

**DOI:** 10.3390/biology15131032

**Published:** 2026-06-28

**Authors:** Annely Zamudio-López, Cristina García-De la Peña, Gerardo Álvarez-Hernández, Sergio I. Barraza-Guerrero, César A. Meza-Herrera, María G. Sánchez-Loera, Edén A. Luna-Zapién, Diana E. Salazar-Nevárez, Javier Carrillo-Campos

**Affiliations:** 1 Conservation Medicine Laboratory, Biological Sciences Faculty, Juarez University of the State of Durango, Gomez Palacio 35010, Dgo., Mexico; annely_zamudio02@hotmail.com; 2 Department of Medicine and Health Sciences, University of Sonora, Hermosillo 83000, Son., Mexico; gerardo.alvarez@unison.mx; 3 Department of Health and Hygiene, Antonio Narro Agrarian Autonomous University, Torreón 27054, Coah., Mexico; loeramg@hotmail.com; 4 Regional University Unit on Arid Lands, Chapingo Autonomous University, Mapimi 35230, Dgo., Mexico; cmeza2020@hotmail.com; 5 Faculty of Chemical Sciences, Juarez University of the State of Durango, Gomez Palacio 35010, Dgo., Mexico; edenareli.luna@ujed.mx; 6 Faculty of Veterinary Medicine and Animal Zootechny, Antonio Narro Agrarian Autonomous University, Torreón 27054, Coah., Mexico; mvz.dianasalazar@gmail.com; 7 Faculty of Zootechnics and Ecology, Autonomous University of Chihuahua, Chihuahua 31453, Chih., Mexico; jccarrillo@uach.mx

**Keywords:** *Rhipicephalus sanguineus*, tick microbiome, ecological conditions, *Coxiella* endosymbionts, *Rickettsia rickettsii*, vector-borne diseases, One Health

## Abstract

Geographic and ecological variations are frequently associated with differences in the microbiota of arthropod vectors, with potential implications for pathogen transmission and public health. This study characterized and compared the bacterial microbiota associated with the brown dog tick (*Rhipicephalus sanguineus*) across three ecologically contrasting regions of Mexico: Cancun (Quintana Roo), Comarca Lagunera (Durango–Coahuila), and Hermosillo (Sonora). Non-engorged ticks collected from stray dogs were analyzed using *16S rRNA gene* (V3–V4) sequencing. Amplicon sequence variants (ASVs) generated in QIIME2 were used for taxonomic, diversity, and predictive functional analyses. Proteobacteria dominated all samples, with *Coxiella*-like bacteria tentatively assigned as *Coxiella mudrowiae* identified as a dominant taxon across all localities. Significant geographic differences were observed in alpha and beta diversity, with Comarca Lagunera showing the highest diversity and Hermosillo the lowest. Sequences tentatively assigned to *Rickettsia rickettsii* were detected exclusively in two pools from Hermosillo. Functional predictions revealed a conserved metabolic repertoire alongside geographic variation in pathway abundance. Overall, the results support the existence of a stable symbiotic component accompanied by a geographically variable bacterial fraction associated with ecologically contrasting regions. These findings highlight the importance of geographic context in shaping tick-associated bacterial communities.

## Simple Summary

Ticks are important carriers of bacteria that can affect both animals and humans. The brown dog tick (*Rhipicephalus sanguineus*) is widely distributed and commonly found in close association with domestic dogs, making it relevant for public health. In this study, we analyzed the bacterial communities associated with this tick in three regions of Mexico with contrasting environmental characteristics: a tropical area (Cancun, Quintana Roo), a semi-arid region (Comarca Lagunera, Durango-Coahuila), and an arid desert environment (Hermosillo, Sonora). We found that tick-associated bacterial communities differed among regions in both diversity and composition. While some bacterial taxa were consistently present across all localities, others showed marked geographic variation in relative abundance. Sequences tentatively assigned to *Rickettsia rickettsii*, the bacterium associated with Rocky Mountain spotted fever, were detected only in ticks from Hermosillo, a region where severe outbreaks of this disease have previously been reported. Overall, these findings indicate that geographically and ecologically contrasting regions are associated with variation in the microbiota of *R. sanguineus*, with potential implications for pathogen circulation and disease ecology. Understanding how tick-associated microbial communities vary across regions may help improve surveillance strategies and support future research within a One Health framework.

## 1. Introduction

Ticks are hematophagous ectoparasites of major medical and veterinary importance due to their role as vectors of a wide diversity of microorganisms affecting both animals and humans [1]. Among them, *Rhipicephalus sanguineus* (Latreille, 1806) sensu lato is particularly relevant because of its close association with domestic dogs, endophilic behavior, and broad distribution across tropical, subtropical, arid, and semi-arid regions, features that facilitate frequent contact with peridomestic environments and increase its epidemiological importance [2,3]. Beyond their role as vectors, ticks are now recognized as complex biological systems that harbor diverse microbial communities composed not only of pathogens, but also of endosymbionts, commensals, and environmental bacteria that may influence host physiology, development, fitness, and vector competence [4,5].

Recent advances in high-throughput sequencing have substantially expanded our understanding of tick-associated microbiota, revealing that these microbial communities are dynamic assemblages influenced by multiple interacting intrinsic and extrinsic factors. These include tick species, sex, life stage, geography, habitat characteristics, host availability, and feeding history [4,5,6,7]. Comparative studies have demonstrated that collection site and geographic region can influence alpha and beta diversity, taxonomic composition, and the relative abundance of dominant bacterial taxa [5,8,9]. Environmental variation may also affect local microclimatic conditions, host assemblages, and habitat suitability for ticks, potentially contributing to shifts in microbial community composition and pathogen circulation [10,11,12].

An additional layer of complexity arises from the fact that non-pathogenic members of the tick microbiome may affect pathogen acquisition, persistence, and transmission. Endosymbionts and other resident bacteria can inhibit or facilitate pathogen colonization, modulate immune processes, and alter vector competence, indicating that the epidemiological significance of ticks cannot be fully understood by focusing exclusively on recognized pathogens [4,5,13,14]. In particular, *Coxiella*-like endosymbionts, related to the genus *Coxiella* (Philip, 1948), have received considerable attention because they are frequently dominant members of the tick-associated microbiota and are thought to contribute to nutritional supplementation and host fitness. At the same time, the abundance of other bacterial genera of medical relevance, including *Rickettsia* (da Rocha-Lima, 1916), *Francisella* (Dorofe’ev, 1947), *Anaplasma* (Theiler, 1910), and *Bartonella* (Strong et al., 1915), may vary according to geography, host identity, and local ecological conditions [4,6,7].

In *R. sanguineus*, studies from different regions of the world have consistently reported the coexistence of conserved bacterial components together with a geographically variable microbial fraction associated with local environmental conditions [15,16,17,18]. However, most available studies have focused primarily on dominant taxa or single populations, whereas fewer have evaluated how environmentally contrasting regions may be associated with differences in microbial diversity, taxonomic structure, and predicted functional profiles within this vector. This knowledge gap is particularly relevant in Mexico, where *R. sanguineus* is recognized as an important vector of public health concern and where ecological conditions vary markedly among regions. Although previous local-scale studies have documented the predominance of *C. mudrowiae* and the presence of bacteria of medical interest in this species [19], broader comparative analyses across contrasting Mexican environments remain limited. Therefore, the aim of this study was to characterize and compare the bacterial microbiota associated with *R. sanguineus* across three ecologically contrasting regions of Mexico (Cancun, Quintana Roo; Comarca Lagunera, Durango–Coahuila; and Hermosillo, Sonora) and to evaluate whether geographically distinct populations exhibit differences in microbial diversity, taxonomic composition, and predicted functional profiles. We hypothesized that populations from environmentally contrasting regions would display distinct microbiome structures and predicted functional potentials, reflecting geographic and ecological variation among regions.

## 2. Materials and Methods

### 2.1. Study Area and Sample Collection

**Figure 1:**
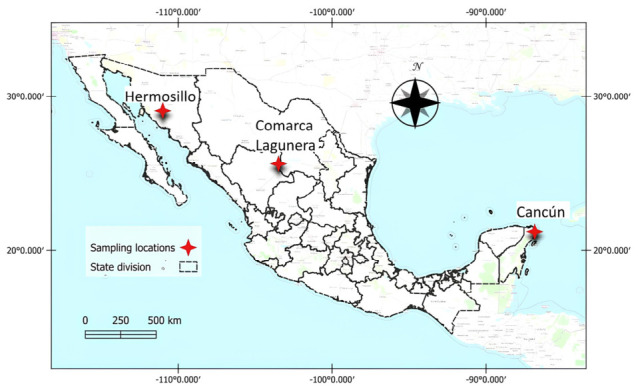
Geographic location of the sampling locations: Cancun, Quintana Roo; Comarca Lagunera, Durango-Coahuila; Hermosillo, Sonora.

The study area included three localities representative of distinct climatic regions in Mexico: Cancun, Quintana Roo (21.18° N, 86.84° W), Comarca Lagunera, Durango-Coahuila (25.60° N, 103.48° W), and Hermosillo, Sonora (29.05° N, 111.00° W) (Figure 1). These locations exhibit contrasting environmental conditions, ranging from a tropical humid climate (Cancun: annual temperature ~26.0–26.5 °C; annual precipitation ~1200–1300 mm), to semi-arid (Comarca Lagunera: ~22.0–24.0 °C; ~200–300 mm), and arid conditions (Hermosillo: ~25.0–26.0 °C; ~300–350 mm) [20].

**Figure 2:**
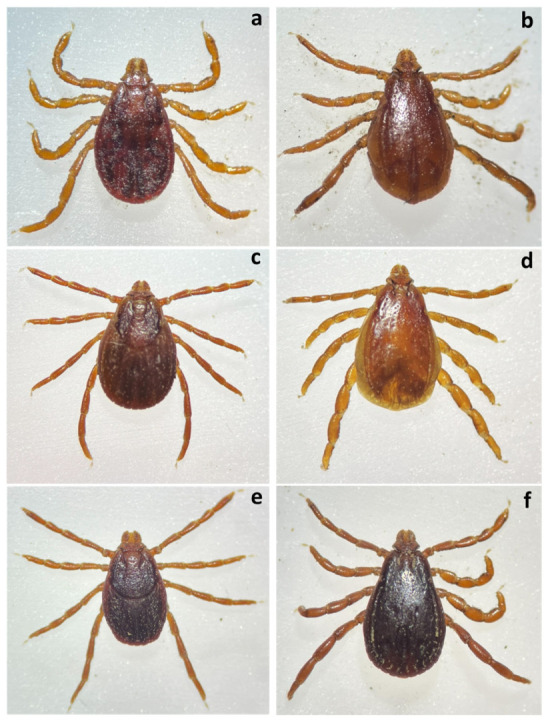
Representative non-engorged adult *Rhipicephalus sanguineus* ticks collected from the three study localities. Cancun, Quintana Roo ((**a**) = female, (**b**) = male); Comarca Lagunera, Durango–Coahuila ((**c**) = female, (**d**) = male); and Hermosillo, Sonora ((**e**) = female, (**f**) = male).

In May 2024, field surveys were conducted across the three localities to identify free-roaming dogs for the collection of adult ticks showing no visible signs of engorgement. This selection criterion was adopted to minimize the potential influence of host blood-derived bacteria on microbiota characterization. Feeding status was assessed based on external morphology, and only specimens lacking the marked abdominal distension and cuticular expansion characteristic of blood-engorged ticks were included in the study (Figure 2). In each locality, ticks were collected from approximately 45–50 dogs. Animals were selected randomly, regardless of age or sex, prioritizing those with visible tick infestations. Tick removal was performed through direct inspection of the ears, dorsum, chest, and extremities. Subsequently, live ticks were placed in sterile plastic containers for transport and further processing in the laboratory.

### 2.2. Laboratory Work

The taxonomic identity of ticks was confirmed using the taxonomic keys of Castillo-Martínez et al. [21]. Only ticks meeting the selection criteria described in Section 2.1 were included in the laboratory analyses. Ticks were first washed with sterile distilled water, followed by immersion in 1% sodium hypochlorite to reduce external bacterial contamination from the exoskeleton, and subsequently rinsed again with sterile purified water to remove residual disinfectant [22]. Subsequently, eight pools per locality were constructed, each consisting of whole internal tick material obtained after transverse sectioning of 25 adult ticks. Ticks were cut transversely under a stereomicroscope using sterile scalpel blades. No specific organs or tissues were isolated prior to DNA extraction. Each pool was placed in a BashingBead™ tube (Zymo Research, Irvine, CA, USA) containing silicon beads and 750 μL of lysis buffer (Zymo Research). Samples were mechanically disrupted using a TerraLyzer™ (Zymo Research) for 30 s to ensure effective tissue maceration.

Bacterial DNA was extracted using the ZymoBIOMICS™ DNA Miniprep Kit (Zymo Research), following the manufacturer’s instructions. All procedures were carried out under sterile conditions in a UV-sterilized laminar flow hood (Thermo Scientific, Marietta, OH, USA). Extracted DNA samples were sent to Novogene Co., Ltd. (Davis, CA, USA) for amplification and sequencing of the V3–V4 region of the *16S rRNA gene*. Universal primers 341F (CCTAYGGGRBGCASCAG) and 806R (GGACTACNNGGGTATCTAAT) were used. PCR reactions were performed in a final volume of 15 μL using Phusion^®^ High-Fidelity PCR Master Mix (New England Biolabs, Ipswich, MA, USA), with 2 μM of each primer and approximately 10 ng of template DNA. Thermal cycling conditions consisted of an initial denaturation at 98 °C for 1 min, followed by 30 cycles of denaturation at 98 °C for 10 s, annealing at 50 °C for 30 s, and extension at 72 °C for 30 s. PCR products were verified by electrophoresis on a 2% agarose gel stained with SYBR™ Green (Thermo Scientific, Waltham, MA, USA). Amplicons generated from each pooled fecal sample were first purified using the QIAquick Gel Extraction Kit (Qiagen™, Hilden, Germany). Sequencing libraries were subsequently prepared individually using the TruSeq DNA PCR-Free Sample Preparation Kit (Illumina, San Diego, CA, USA), incorporating unique sample-specific indices (barcodes) to enable post-sequencing demultiplexing. Library quality and concentration were assessed using a Qubit 2.0 fluorometer (Thermo Scientific) and an Agilent Bioanalyzer 2100 (Agilent Technologies, Santa Clara, CA, USA). Indexed libraries were then normalized and pooled at equimolar concentrations prior to sequencing on an Illumina NovaSeq 6000 platform (Illumina), generating paired-end reads of 250 bp.

### 2.3. Bioinformatic Analysis

Sequence processing was performed in QIIME2 (version 2023.5) within a Linux–Ubuntu environment [23]. Raw paired-end reads were denoised using the DADA2 pipeline [24] to remove low-quality sequences and chimeras, and to infer amplicon sequence variants (ASVs). Sequencing depth was assessed using Good’s coverage [25], with values between 0.95 and 1.00 considered indicative of adequate sampling completeness. Taxonomic classification was performed using a Naïve Bayes classifier trained on the Greengenes2 reference database [26]. Species-level assignments were tentatively inferred using BLAST+ (version 2.17.0) under stringent criteria (E-value = 0.0, ≥98% sequence identity, and 100% query coverage). To minimize ambiguity in species-level assignments, only BLAST matches meeting these criteria and yielding a single unambiguous best hit were accepted. In cases where multiple species shared equivalent sequence identity and query coverage values, species-level identification was not attempted and taxonomic assignment was conservatively retained at the genus level [27]. Heatmaps representing relative abundances were generated using BioRender.com and Adobe Illustrator 2025 (Ver. 29.0).

To standardize sequencing depth across samples, rarefaction was applied prior to diversity analyses. Alpha diversity metrics, including observed ASVs, Shannon entropy, Pielou’s evenness, and Faith’s phylogenetic diversity, were calculated. Differences among localities were evaluated using Kruskal–Wallis tests (*p* < 0.05). When significant differences were detected, pairwise comparisons were performed using Dunn’s post hoc test (*p* < 0.05). Significant results were visualized using boxplots in GraphPad Prism (version 8.0.2). Beta diversity was assessed using Jaccard, Bray–Curtis, unweighted UniFrac, and weighted UniFrac distance metrics. Statistical differences in community composition among localities were evaluated using permutational multivariate analysis of variance (PERMANOVA) (*p* < 0.05). Principal coordinate analysis (PCoA) plots were generated using RStudio ver. 2024.12.1 [28]. To identify taxa contributing to differences among localities, similarity percentage analysis (SIMPER) was performed at the phylum and species levels using PAST 5.3 (University of Oslo, Norway). Taxa with SIMPER contributions greater than 0.1% were further evaluated among localities using Kruskal–Wallis tests (*p* < 0.05). Taxa showing significant differences were visualized using heatmaps constructed from the mean relative abundance of each taxon within each locality. To facilitate comparison among localities, mean values were standardized using z-score transformation. Taxa were ranked according to their percentage contribution to community dissimilarity (DC) as determined by SIMPER analysis. Positive z-scores indicate relative enrichment, whereas negative z-scores indicate relative depletion compared with the overall mean abundance across localities. Heatmaps were generated using BioRender.com and Adobe Illustrator.

### 2.4. Functional Predictions Using PICRUSt2

Functional potential of the bacterial microbiota associated with *R. sanguineus* was inferred using PICRUSt2 (v2.5.2) (Phylogenetic Investigation of Communities by Reconstruction of Unobserved States) [29], based on *16S rRNA gene* sequences (V3–V4 region) previously processed into amplicon sequence variants (ASVs). Prediction accuracy was assessed using the Nearest Sequenced Taxon Index (NSTI), which quantifies the phylogenetic distance between sampled organisms and reference genomes. Lower NSTI values indicate higher prediction reliability [29]. The PICRUSt2 pipeline included phylogenetic placement of ASVs into a reference tree using EPA-NG and GAPPA, followed by hidden-state prediction of gene family abundances implemented in the castor R package. Predicted gene families were subsequently annotated into KEGG Orthologs (KOs) and used to reconstruct metabolic pathways based on the MetaCyc database. To normalize data distribution, predicted pathway abundances were log-transformed (log1p). Differences in pathway abundances among localities were tested using Kruskal–Wallis tests (*p* < 0.05), with false discovery rate (FDR) correction applied for multiple comparisons. Pathways with significant differences were further analyzed through pairwise comparisons using Wilcoxon rank-sum tests (*p* < 0.05, FDR-adjusted). To assess the magnitude and direction of functional differences, median differences (Δ median) between localities were calculated. For heatmap visualization, pathways were ranked according to the magnitude of variation among localities, calculated as the difference between the maximum and minimum median relative abundance values (delta range). The 30 pathways exhibiting the greatest delta range were selected for visualization. Median pathway abundances were subsequently standardized using z-score transformation to facilitate comparisons among localities. Positive z-scores indicate relative enrichment, whereas negative z-scores indicate relative depletion relative to the overall median abundance across localities. Pairwise functional differences were visualized using volcano plots based on Δ median values and FDR-adjusted q-values. Heatmaps were generated using BioRender.com, whereas volcano plots were produced in RStudio.

## 3. Results

### 3.1. Sequencing Output and Taxonomic Composition

The average number of sequencing reads obtained for ticks from Cancun, Comarca Lagunera, and Hermosillo was 205,935.9, 190,754.3, and 206,635.0, respectively. After quality filtering and chimera removal, the average number of non-chimeric sequences was 184,521.1 (89.60%) for Cancun, 159,379.7 (82.36%) for Comarca Lagunera, and 186,618.3 (90.32%) for Hermosillo (Table S1). A total of eight pools were initially constructed per locality; however, two pools from Comarca Lagunera did not yield sufficient DNA or sequencing output and were excluded from downstream analyses. Therefore, six pools from Comarca Lagunera were included in the final dataset. Good’s coverage values ranged from 0.999 to 1.000 across all samples (Table S1), indicating that sequencing depth was sufficient to capture the majority of bacterial diversity present in each sample.

Bacterial taxonomic richness at each taxonomic level per locality is summarized in Table 1 and detailed in Table S2. Following BLAST-based taxonomic assignment against the NCBI database, a total of 63 tentatively assigned bacterial species were identified in Cancun, 220 in Comarca Lagunera, and 53 in Hermosillo.

**Figure 3:**
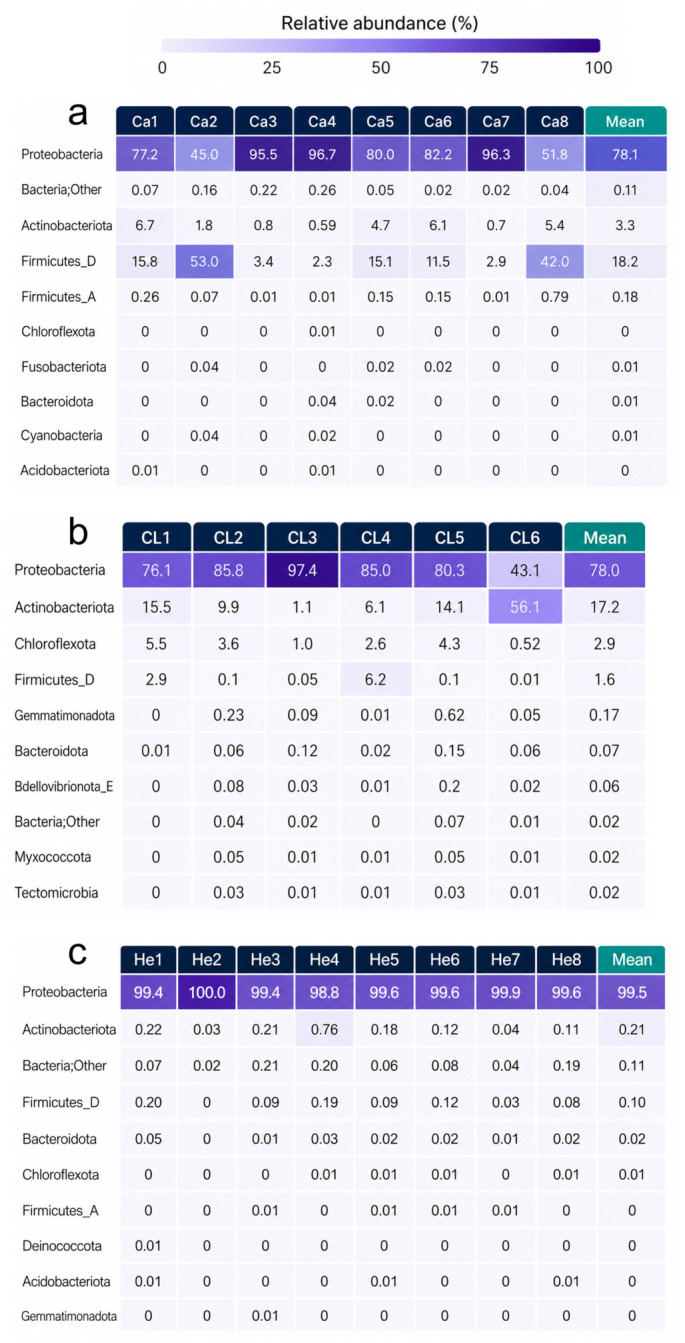
Phylum-level heatmaps showing the 10 most abundant bacterial phyla detected in *Rhipicephalus sanguineus* ticks from three Mexican localities: Cancun (**a**), Comarca Lagunera (**b**), and Hermosillo (**c**).

**Figure 4:**
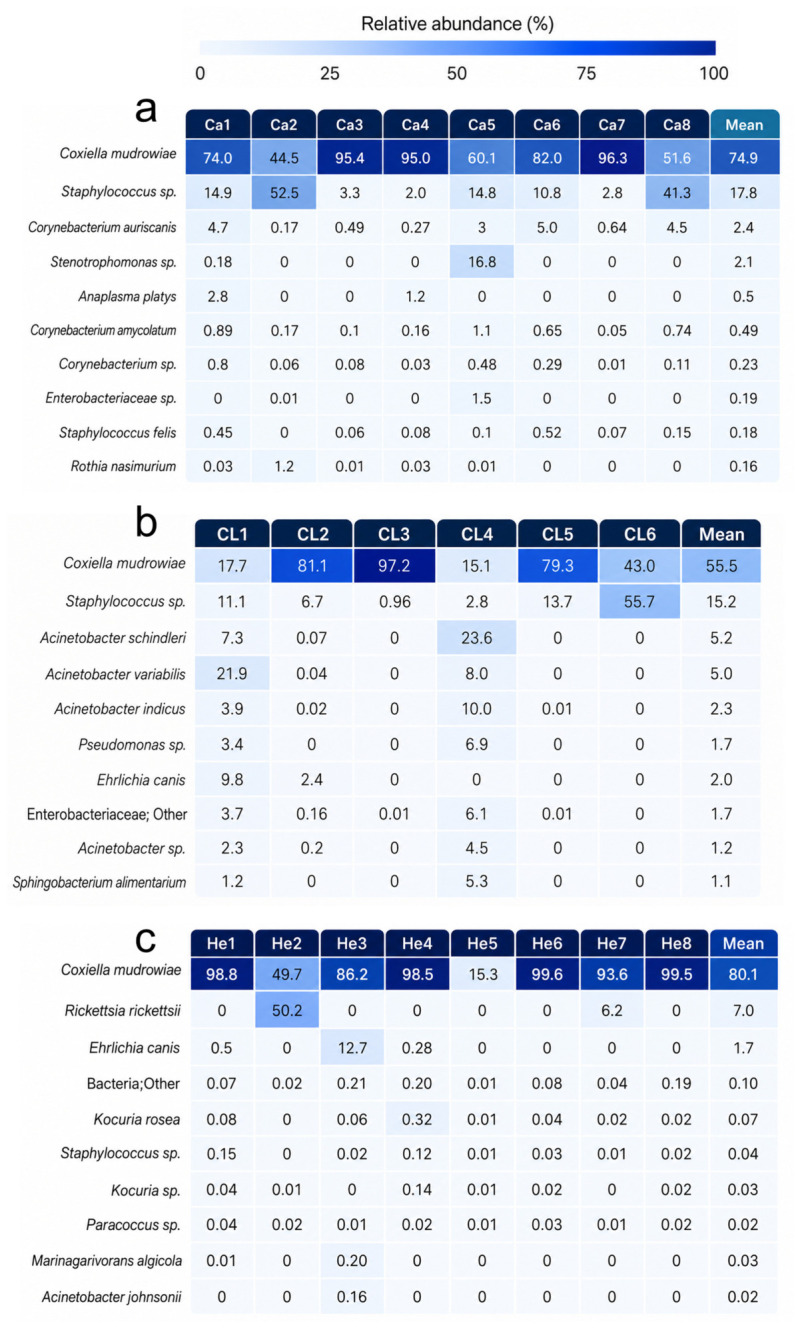
Species-level heatmaps showing the 10 most abundant bacterial species detected in *Rhipicephalus sanguineus* ticks from three Mexican localities: Cancun (**a**), Comarca Lagunera (**b**), and Hermosillo (**c**).

At the phylum level, Proteobacteria dominated the bacterial communities in ticks from all three localities (Cancun: mean = 78.10%; Comarca Lagunera: mean = 77.95%; Hermosillo: mean = 99.54%) (Figure 3a–c). At the species level, *Coxiella*-like bacteria (tentatively assigned as *Coxiella mudrowiae*) were the most abundant taxa in ticks from Cancun and Comarca Lagunera, followed by an unidentified *Staphylococcus* species (Cancun: mean = 17.86%; Comarca Lagunera: mean = 15.18%) (Figure 4a,b). In Hermosillo, *C. mudrowiae* was also the dominant taxon (mean = 80.13%). Additionally, sequences tentatively assigned to *Rickettsia rickettsii* were detected exclusively in two of the eight analyzed pools from Hermosillo (50.2% and 6.2%, respectively), whereas no sequences assigned to this species were detected in Cancun or Comarca Lagunera (Figure 4c).

### 3.2. Alpha Diversity

**Figure 5:**
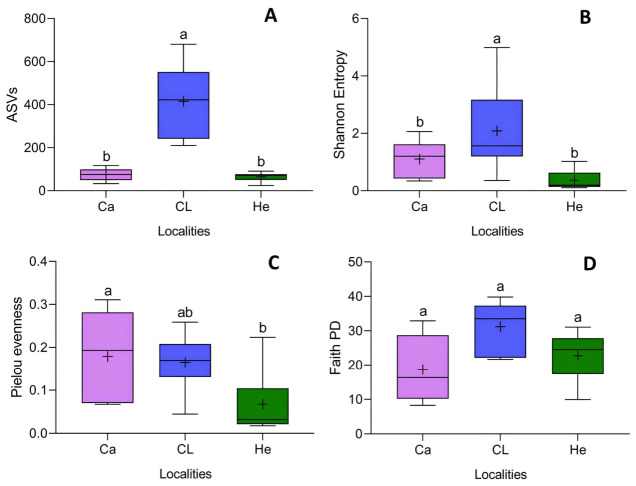
Boxplots showing alpha diversity metrics of the bacterial microbiota associated with *Rhipicephalus sanguineus* ticks from three localities in Mexico (Ca = Cancun, CL = Comarca Lagunera, He = Hermosillo). (**A**) Observed ASVs (richness); (**B**) Shannon diversity index; (**C**) Pielou’s evenness; and (**D**) Faith’s phylogenetic diversity (Faith PD). The horizontal line within each box represents the median, and the “+” symbol indicates the mean. Different letters above the boxplots indicate statistically significant differences among localities according to the Kruskal–Wallis test followed by Dunn’s post hoc test (*p* < 0.05).

Significant differences were observed in three alpha diversity metrics evaluated, indicating variation in bacterial richness, diversity, and evenness among localities. Samples from Comarca Lagunera exhibited higher bacterial richness and diversity compared to Cancun and Hermosillo. Observed features differed significantly among localities (Kruskal–Wallis H = 11.23, *p* < 0.001), with mean values of 75.50 for Cancun, 429.0 for Comarca Lagunera, and 65.13 for Hermosillo (Figure 5A). Similarly, the Shannon diversity index showed significant differences among localities (H = 9.25, *p* < 0.001), with mean values of 1.11 for Cancun, 1.99 for Comarca Lagunera, and 0.37 for Hermosillo (Figure 5B). Significant differences were also observed in Pielou’s evenness index (H = 6.85, *p* = 0.026), with Cancun showing the highest mean value (0.17) and Hermosillo the lowest (0.06) (Figure 5C). In contrast, Faith’s phylogenetic diversity did not differ significantly among localities (H = 3.72, *p* = 0.157), with mean values of 18.73, 30.18, and 22.77 for Cancun, Comarca Lagunera, and Hermosillo, respectively (Figure 5D).

### 3.3. Beta Diversity

**Figure 6:**
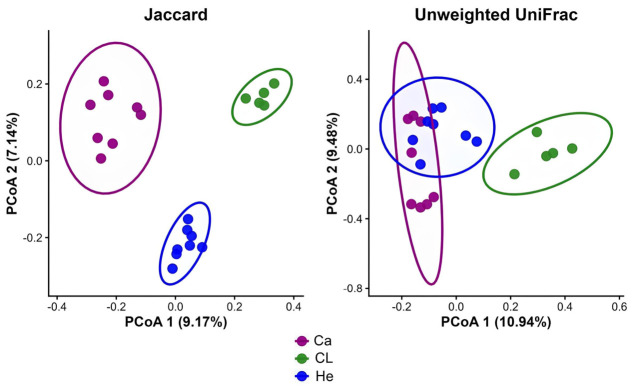
Principal coordinates analysis (PCoA) plots based on the Jaccard index and unweighted UniFrac of the bacterial microbiota of *Rhipicephalus sanguineus* ticks from three localities in Mexico: Cancun (Ca), Comarca Lagunera (CL), and Hermosillo (He).

Beta diversity analyses revealed significant differences in bacterial community composition among localities for three of the four metrics evaluated. PERMANOVA tests indicated significant differences for the Jaccard index (pseudo-F = 1.708, *p* = 0.001), Bray–Curtis dissimilarity (pseudo-F = 2.333, *p* = 0.018), and unweighted UniFrac (pseudo-F = 1.824, *p* = 0.001). In contrast, the weighted UniFrac metric did not show significant differences among localities (pseudo-F = 2.353, *p* = 0.056). Clear clustering by locality was observed in PCoA ordinations based on Jaccard and unweighted UniFrac distances (Figure 6).

### 3.4. Taxa Contributing to Community Dissimilarity

**Figure 7:**
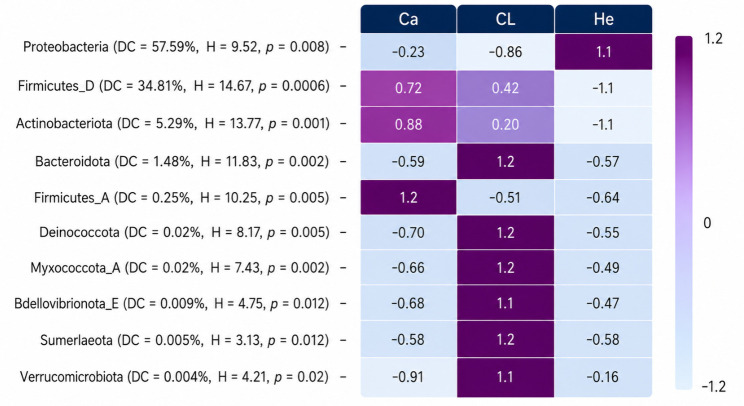
Phylum-level heatmap showing the bacterial microbiota of *Rhipicephalus sanguineus* ticks from three localities in Mexico (CA = Cancun, CL = Comarca Lagunera, HE = Hermosillo) highlighting the taxa that contributed significantly to community dissimilarity among localities. Taxa were ranked according to their contribution to community dissimilarity (DC) and visualized using z-score standardized locality means. Positive z-scores indicate means enriched relative to the overall mean abundance, whereas negative z-scores indicate relative depletion. H = Kruskal–Wallis statistic, *p* = significance level.

**Figure 8:**
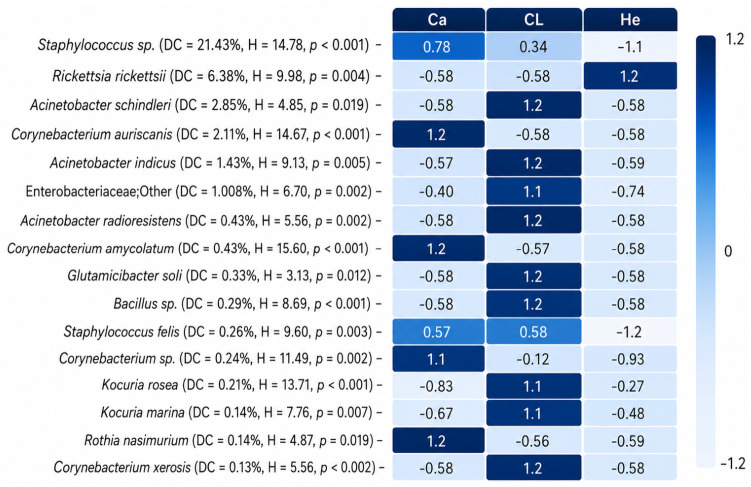
Species-level heatmap showing the bacterial microbiota of *Rhipicephalus sanguineus* ticks from three localities in Mexico (CA = Cancun, CL = Comarca Lagunera, HE = Hermosillo) highlighting the taxa that contributed significantly to community dissimilarity among localities. Taxa were ranked according to their contribution to community dissimilarity (DC) and visualized using z-score standardized locality means. Positive z-scores indicate means enriched relative to the overall mean abundance, whereas negative z-scores indicate relative depletion. H = Kruskal–Wallis statistic, *p* = significance level.

SIMPER analysis revealed an overall dissimilarity of 25.77% at the phylum level among localities. Eleven phyla differed significantly in relative abundance among Cancun, Comarca Lagunera, and Hermosillo (Figure 7). Proteobacteria contributed the most to this dissimilarity, followed by Firmicutes_D and Actinobacteriota. At the species level, overall dissimilarity increased to 40.33%, with significant differences detected in seventeen bacterial taxa. *Staphylococcus* sp. contributed the most to community dissimilarity, followed by sequences tentatively assigned to *Rickettsia rickettsii* and *Acinetobacter schindleri* (Figure 8).

### 3.5. Functional Profile of Predicted Metabolic Pathways

Functional prediction using PICRUSt2 identified a total of 424 metabolic pathways, including 388 detected in Cancun, 416 in Comarca Lagunera, and 384 in Hermosillo (Table S3). NSTI values were consistently low across all samples (means: Cancun = 0.00485, Comarca Lagunera = 0.00679, and Hermosillo = 0.00405), indicating high phylogenetic similarity to reference genomes and supporting the relative reliability of PICRUSt2 functional predictions. The most abundant predicted metabolic pathways revealed broadly similar functional profiles across the three localities. On average, the five most abundant pathways corresponded primarily to central metabolic processes, including aerobic respiration I (cytochrome c), gondoate biosynthesis (anaerobic), cis-vaccenate biosynthesis, the tricarboxylic acid (TCA) cycle I (prokaryotic), and adenosine deoxyribonucleotides de novo biosynthesis II. These pathways are associated with essential bacterial metabolic functions, particularly energy production, lipid biosynthesis, and nucleotide synthesis, suggesting that they represent the conserved predicted metabolic repertoire of the bacterial communities associated with ticks. Although the overall predicted functional profiles were broadly similar among the three localities, some variability in relative pathway abundance was observed among individual samples.

### 3.6. Functional Differentiation Among Localities

**Figure 9:**
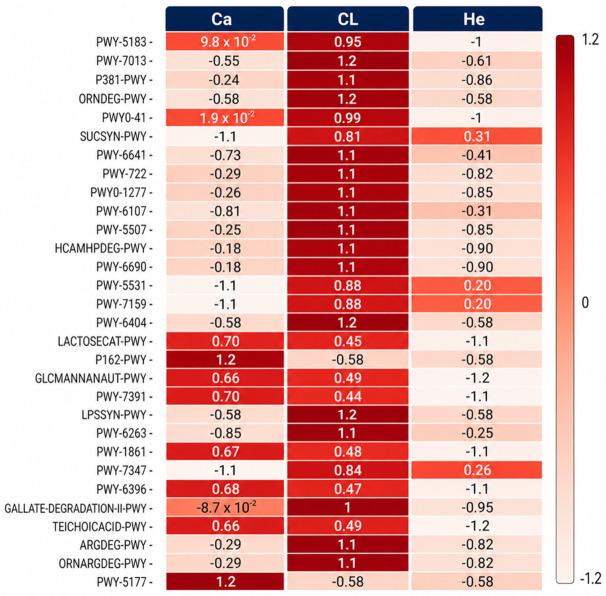
Heatmap of the 30 predicted MetaCyc pathways exhibiting the greatest variation among tick samples from Cancun, Comarca Lagunera, and Hermosillo. Pathways were ranked according to the delta range (maximum minus minimum locality median abundance) and visualized using z-score standardized median abundances. Positive z-scores indicate pathways enriched relative to the overall median abundance, whereas negative z-scores indicate relative depletion. Detailed pathway descriptions are provided in Table S3.

A total of 298 predicted metabolic pathways differed significantly in relative abundance among the three localities (q < 0.05). To further explore these differences, the most divergent predicted metabolic pathways were visualized using a heatmap based on the median relative abundance of each pathway per locality (Figure 9). Pathways were ranked according to the magnitude of variation among localities (delta range) and standardized using a z-score transformation to facilitate comparison. The heatmap revealed clear differences in the relative abundance patterns of several predicted MetaCyc pathways across the three geographic regions. Many pathways exhibited higher predicted relative abundance in ticks from Comarca Lagunera, whereas the same pathways tended to show comparatively lower values in Hermosillo. Samples from Cancun generally displayed intermediate abundance patterns across multiple pathways. Overall, these results suggest that a subset of predicted metabolic functions varies among localities, indicating geographic variation in predicted functional potential.

### 3.7. Pairwise Comparison of Functional Pathways

Pairwise comparisons between localities revealed multiple predicted metabolic pathways that differed significantly in relative abundance (Wilcoxon rank-sum test with FDR correction, q < 0.05). The comparison between Cancun and Comarca Lagunera showed 47 pathways significantly enriched in Comarca Lagunera, whereas no pathways were strongly enriched in Cancun. In contrast, the comparison between Cancun and Hermosillo revealed 118 pathways enriched in Cancun and only 10 pathways enriched in Hermosillo. Similarly, the comparison between Comarca Lagunera and Hermosillo indicated a large number of pathways enriched in Comarca Lagunera (284) relative to Hermosillo. These patterns are consistent with the functional differentiation observed in the heatmap analysis (Figure 9) and further support geographic variation in predicted metabolic profiles among localities. Detailed results of pairwise comparisons are provided in Supplementary Figure S1.

## 4. Discussion

### 4.1. Ecological Context

The present study revealed marked geographic variation in the bacterial microbiota associated with *R. sanguineus* across populations from Cancun, Comarca Lagunera, and Hermosillo. Despite belonging to the same tick species, these populations exhibited significant differences in taxonomic composition, diversity patterns, and predicted metabolic potential, supporting the idea that tick-associated microbiota may vary according to ecological context [8,10]. These findings are consistent with previous studies showing that microbiota composition in ticks is associated with both intrinsic and extrinsic factors, including species identity, life stage, sex, geography, habitat characteristics, and microclimatic conditions [4,5,6,13]. In particular, recent studies have identified collection site and geographic region as important correlates of microbial community composition in ticks, in some cases exceeding the influence of host-related variables [6].

From an ecological perspective, the observed differences in presence/absence-based beta diversity metrics (Jaccard and unweighted UniFrac) suggest that variation among populations may be primarily associated with turnover in less abundant taxa, a pattern consistent with environmentally associated microbial assembly processes [30]. Such patterns are frequently interpreted as consistent with environmental filtering, in which abiotic conditions may favor taxa capable of persisting under specific ecological constraints [30,31]. However, these interpretations should be considered cautiously, since environmental variables were not directly measured in the present study. Habitat modification, vegetation shifts, fragmentation, and climate-driven changes in tick distribution have previously been associated with alterations in microbial community composition and pathogen circulation within arthropod vectors [10,11,12]. These processes may influence not only where ticks occur, but also which microbial assemblages—including potential pathogens—persist within their microbiota.

The microbiota of *R. sanguineus* exhibited a dual organizational structure, characterized by a conserved symbiotic component dominated by *Coxiella*-like bacteria tentatively assigned as *C. mudrowiae*, together with a geographically variable bacterial fraction. The predominance of *Coxiella*-like endosymbionts is consistent with previous reports across diverse geographic regions and reflects their recognized importance in nutritional supplementation and host fitness. These endosymbionts are thought to contribute to B-complex vitamin synthesis and may play a central role in tick physiology, particularly under nutrient-limited conditions associated with hematophagy [4,32,33,34]. In contrast, the variable fraction of the microbiota displayed marked geographic differentiation. The higher diversity observed in Comarca Lagunera may be associated with greater environmental heterogeneity and exposure to diverse microbial reservoirs, whereas the lower diversity detected in Hermosillo may be associated with the extreme ecological conditions characteristic of desert environments. Overall, these patterns are consistent with the idea that abiotic and ecological gradients may contribute to variation in tick-associated microbial communities [8,30,31].

At the genera level, the co-occurrence of environmental bacteria and potentially pathogenic taxa, including *Rickettsia*, *Staphylococcus*, and *Acinetobacter*, suggests a complex microbial network within the tick microbiome. Increasing evidence indicates that microbial interactions may influence pathogen establishment and persistence through mechanisms involving competition, facilitation, or immune modulation [4,14]. Functional predictions further supported this ecological framework. Although central metabolic pathways remained relatively conserved, variation in predicted pathway abundance among populations suggests that taxonomic differences may also be associated with differences in inferred metabolic potential. Previous studies have also suggested that environmental variation may be associated with differences in microbial composition and predicted functional capacity, with potential implications for vector competence and pathogen transmission [4,29]. Overall, these findings are consistent with a conceptual framework in which the microbiome of *R. sanguineus* consists of a stable symbiotic component accompanied by a geographically variable bacterial fraction potentially associated with local ecological conditions. However, this framework should be interpreted as an ecological model consistent with the observed data rather than as definitive evidence of direct environmental causality.

### 4.2. Public Health Context

The findings of this study acquire particular relevance within a public health context when considering the marked ecological differences among the three analyzed regions (Cancun, Comarca Lagunera, and Hermosillo), which differ substantially in climate, vegetation, and environmental conditions. These ecological contrasts have been associated with variation in tick ecology and with differences in the structure of their associated microbiome, which may in turn be relevant to pathogen transmission dynamics.

The detection of sequences tentatively assigned to *R. rickettsii* in two pools from Hermosillo is epidemiologically relevant because this pathogen is the etiological agent of Rocky Mountain spotted fever (RMSF), one of the most severe tick-borne diseases in the Americas and a major public health problem in northern Mexico [35,36]. Epidemiological studies have shown that northern states such as Sonora exhibit some of the highest case-fatality rates reported worldwide, reaching values up to 37.9%, substantially exceeding those observed in many other endemic regions [35,37]. These findings further support the epidemiological relevance of monitoring *R. rickettsii* circulation within local tick populations. From an ecological perspective, the environmental conditions characteristic of Hermosillo, including high temperatures, low humidity, and arid vegetation, may contribute to microbial simplification within tick-associated communities. Reduced microbial diversity could potentially influence microbial interactions within the tick microbiome; however, the present data do not allow direct evaluation of whether these processes affect the persistence of *Rickettsia*. Similar ecological patterns have been described in other endemic regions, where environmental conditions associated with vector ecology may influence disease risk [10,11,12]. In contrast, although cases of rickettsiosis have also been reported in the Comarca Lagunera region, the epidemiological impact has generally been lower compared with northern foci such as Sonora. This difference may reflect regional variation in environmental conditions, host availability, vector ecology, and microbiome structure, all of which could influence pathogen prevalence and transmission dynamics. Indeed, regional differences in case-fatality rates and disease incidence across northern Mexico further support the importance of ecological context in shaping disease dynamics [38].

In the case of Cancun and the broader Yucatán Peninsula, the absence of *Rickettsia* detection in the analyzed non-engorged ticks should not be interpreted as evidence of absence of rickettsial circulation. Increasing evidence indicates that rickettsioses are endemic in southeastern Mexico, where multiple *Rickettsia* species, including *R. rickettsii*, *R. felis*, *R. parkeri*, and *R. typhi*, have been documented in humans, domestic animals, and arthropod vectors [39,40,41]. Previous studies conducted in Yucatán have detected *Rickettsia* spp. in *R. sanguineus*, *Amblyomma mixtum*, and *Ixodes affinis* collected from domestic animals, supporting the existence of active transmission cycles in the region [42]. Likewise, recent epidemiological investigations have reported high seroprevalence rates and strong associations between rickettsial exposure and environmental or sociodemographic factors in tropical communities of southeastern Mexico [43]. Therefore, the lack of detection observed in the present study may reflect local ecological heterogeneity, temporal fluctuations in pathogen circulation, low bacterial prevalence, or limitations associated with sample size and non-engorged tick sampling, rather than the absence of epidemiological risk.

**Figure 10:**
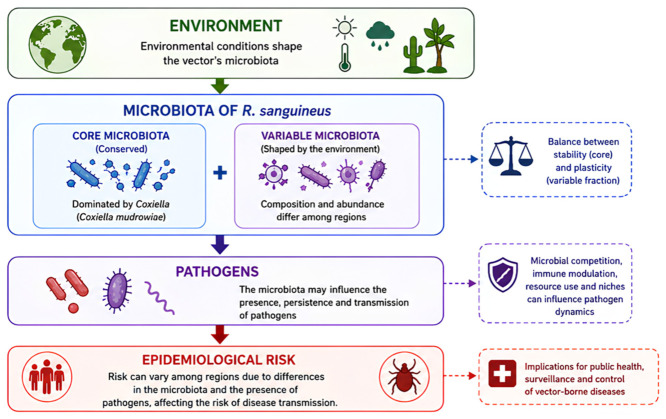
Conceptual framework linking environmental variation, tick microbiota, pathogen dynamics, and public health implications in *Rhipicephalus sanguineus*. Arrows indicate the conceptual relationships among components, whereas colors are used only to visually distinguish the different conceptual modules.

Importantly, the detection of sequences tentatively assigned to *R. rickettsii* in non-engorged ticks from Hermosillo suggests that the pathogen may persist within the vector independently of recent blood feeding, potentially through intracellular maintenance and vertical transmission. Such mechanisms have previously been described for rickettsial pathogens and are considered important for pathogen persistence in endemic regions [36,44]. This observation also raises the possibility that ticks not actively feeding may contribute to pathogen maintenance within endemic transmission cycles. Beyond pathogen detection itself, the broader microbial context in which *Rickettsia* occurs may also be epidemiologically relevant. In this sense, environmentally associated shifts in microbiome structure could indirectly influence pathogen persistence and transmission dynamics. Overall, these findings highlight that the risk associated with tick-borne diseases should not be interpreted solely in terms of vector presence or pathogen detection, but rather as the result of complex interactions among environmental conditions, microbiome structure, vector ecology, and pathogen circulation (Figure 10). Within this framework, the microbiome of *R. sanguineus* may represent an important component in the ecology and epidemiology of rickettsial diseases, with potential implications for surveillance, ecological risk assessment, and the development of targeted control strategies under a One Health perspective [4,45].

Beyond the detection of specific pathogens, the present results suggest that environmentally contrasting regions may be associated with distinct epidemiological scenarios through their effects on microbiome structure and functionality. The marked geographic variation observed in predicted metabolic pathways suggests that differences among environmentally contrasting regions may be associated not only with microbial composition but also with the predicted functional capacity of the bacterial communities. Such differences may influence microbial interactions, resource utilization, and the ecological context in which pathogens persist within vector populations. Although direct causal relationships cannot be established from the present data, these findings support the hypothesis that environmental context and microbiome organization may contribute to shaping pathogen persistence and transmission dynamics across regions. Consequently, evaluating microbiome functionality in addition to taxonomic composition may provide a more comprehensive understanding of vector-borne disease ecology.

### 4.3. Limitations of the Study

Despite the relevant findings obtained in this study, several methodological limitations should be considered when interpreting the results. First, *16S rRNA gene* sequencing enables the characterization of bacterial community composition but presents limitations in taxonomic resolution at the species level and does not provide direct functional evidence. Although conservative taxonomic assignment criteria were applied, some identifications may remain restricted to higher taxonomic levels, potentially limiting species-level resolution for closely related taxa. Furthermore, relative abundance derived from metabarcoding data does not necessarily reflect absolute abundance or biological activity of microorganisms. Another important limitation is the use of pooled tick samples for microbiota analysis. While this strategy provides a population-level overview and reduces experimental costs, it may mask individual variability, including rare infections or heterogeneous colonization patterns. Consequently, results should be interpreted primarily at the population level rather than at the level of individual ticks.

An additional limitation is that environmental variables were not directly measured at the sampling sites. The ecological interpretation of the observed geographic patterns is based on the well-recognized environmental differences among the study regions rather than on site-specific measurements of factors such as temperature, humidity, vegetation structure, or soil characteristics Consequently, although differences in microbiota composition and predicted functional profiles were associated with ecologically contrasting regions, the present study does not allow direct evaluation of causal relationships between environmental conditions and microbiome structure. Future studies integrating environmental metadata with multivariate ecological analyses would help clarify the mechanisms underlying the geographic patterns observed in this study.

Additionally, this study focused exclusively on non-engorged ticks to minimize the detection of host-derived bacteria. However, this approach limits the understanding of microbiome dynamics associated with blood feeding, which has been associated with shifts in microbial composition and the proliferation of certain pathogens, including *Rickettsia*. Future studies incorporating different physiological states of the tick would provide a more comprehensive view of these processes. A further limitation is that two pools from Comarca Lagunera were excluded because of insufficient DNA quality, resulting in a slightly smaller sample size for this locality (*n* = 6) compared with Cancun and Hermosillo (*n* = 8). Although this imbalance was relatively minor, it should be acknowledged as a potential source of reduced statistical power in some comparative analyses and considered when interpreting locality-level patterns.

Particular caution is warranted when interpreting the sequences tentatively assigned to *R. rickettsii*. Although conservative taxonomic assignment criteria were applied, species-level identification based on 16S rRNA amplicons should be interpreted cautiously because closely related taxa may not always be fully resolved. Moreover, the detection of *R. rickettsii*-like sequences in non-engorged ticks does not allow discrimination between viable, active, or dormant bacterial states and therefore cannot provide direct evidence regarding pathogen transmission potential. These sequences were detected exclusively in two of the eight pooled samples analyzed from Hermosillo and were not detected in pools from Cancun or Comarca Lagunera. Because each pool contained 25 ticks, the prevalence of infection at the individual tick level could not be determined. Therefore, the present findings should be interpreted as evidence of the occurrence of sequences tentatively assigned to *Rickettsia rickettsii* within the Hermosillo population rather than as an estimate of infection frequency, prevalence, or epidemiological risk. Additional analyses based on individual ticks and pathogen-specific molecular assays, such as species-specific qPCR, would be necessary to confirm taxonomic identity and determine the true prevalence and distribution of *R. rickettsii* within local tick populations.

Finally, functional predictions generated using PICRUSt2 are based on phylogenetic inference rather than direct detection of genes or metabolic pathways. While this tool provides a useful approximation of functional potential, results should be interpreted cautiously, as predicted pathways do not necessarily reflect actual metabolic activity. In this regard, approaches based on shotgun metagenomics or metatranscriptomics would help validate and refine these functional inferences.

## 5. Conclusions

This study identified geographic structuring in the bacterial microbiota associated with *R. sanguineus* across environmentally contrasting regions of Mexico. Despite belonging to the same tick species, populations from Cancun, Comarca Lagunera, and Hermosillo showed significant differences in taxonomic composition, diversity patterns, and predicted metabolic potential, supporting an association between ecological context and microbiome variation in arthropod vectors. The findings are consistent with a dual organizational model in which a conserved symbiotic component dominated by *C. mudrowiae* coexists with a variable bacterial fraction potentially associated with local ecological conditions. This structure may reflect a balance between symbiotic stability and ecological plasticity, emphasizing the adaptive capacity of the tick microbiome across heterogeneous environments.

Overall, this study highlights the importance of integrating ecological, microbiological, and epidemiological perspectives to better understand the complexity of vector-associated microbiomes. In this context, the microbiome of *R. sanguineus* may represent an active ecological component associated with pathogen ecology, rather than simply a passive reservoir of microorganisms, with important implications for surveillance, ecological risk assessment, and control strategies within a One Health framework. Future studies incorporating shotgun metagenomics, metatranscriptomics, and absolute quantification approaches will be essential to validate functional predictions and to better understand the interactions among symbionts, pathogens, and their arthropod hosts.

## Figures and Tables

**Table 1:** Number of bacterial taxa identified at each taxonomic level across localities.

Localities	Phylum	Class	Order	Family	Genus	Species	STA (BLAST)
Ca	12	18	62	93	165	196	63
CL	25	50	157	257	512	663	220
He	17	28	83	116	190	207	53

Cancun (Ca), Comarca Lagunera (CL), Hermosillo (He), species tentatively assigned based on BLAST analysis STA (BLAST).

## Data Availability

The raw data supporting the conclusions of this article are available in the NCBI Sequence Read Archive (SRA) under accession number PRJNA1455821.

## Supplementary Materials

The following supporting information can be downloaded at: https://www.mdpi.com/article/10.3390/biology15131032/s1, Table S1: Sequencing processing statistics for *16S rRNA gene* (V3–V4) data obtained from *Rhipicephalus sanguineus* samples from three Mexican localities, including read counts before and after filtering, denoising, chimera removal, Good’s coverage, and NSTI values. Table S2: Tentative species-level assignments inferred by BLAST analysis for bacterial taxa detected in *Rhipicephalus sanguineus* microbiota across three Mexican localities. Query coverage, E-values, sequence identity (Per. Ident), accession numbers, and sample abundances are provided. Ca = Cancun, CL = Comarca Lagunera, He = Hermosillo. Table S3: Predicted MetaCyc metabolic pathways identified by PICRUSt2 across individual tick samples from Cancun (Ca), Comarca Lagunera (CL), and Hermosillo (He). Values represent relative pathway abundances for each sample and locality mean values. Figure S1: Volcano plots showing pairwise comparisons of predicted metabolic pathways among localities. Cancun (Ca), Comarca Lagunera (CL), Hermosillo (He). Each point represents a predicted MetaCyc pathway. The *x*-axis indicates the difference in median log-transformed abundance (Δ median), and the *y*-axis represents statistical significance (−log10 FDR-adjusted q-value). Colored points indicate significantly enriched pathways (q < 0.05).

## Abbreviations

The following abbreviations are used in this manuscript:

DNA	Deoxyribonucleic acid
rRNA	Ribosomal ribonucleic acid
NGS	Next-generation sequencing
QIIME2	Quantitative Insights into Microbial Ecology
ASVs	Amplicon sequence variants
BLAST	Basic Local Alignment Search Tool
PCoA	Principal coordinate analysis
PERMANOVA	Permutational multivariate analysis of variance
SIMPER	Similarity percentage analysis
PICRUSt	Phylogenetic Investigation of Communities by Reconstruction of Unobserved States
NSTI	Nearest Sequenced Taxon Index
