# Supplementary material for: Geographic Variation in the Bacterial Microbiota of Rhipicephalus sanguineus (Acari, Ixodidae) Across Environmentally Contrasting Regions of Mexico
Source: Biology (Basel). 2026 Jun 28;15(13):1032. doi: 10.3390/biology15131032 (PMC13360058; doi:10.3390/biology15131032)
Supplement: Supplementary file 1 [file biology-15-01032-s001.zip › Supplementary Figure S1.pdf]

Supplementary Figure

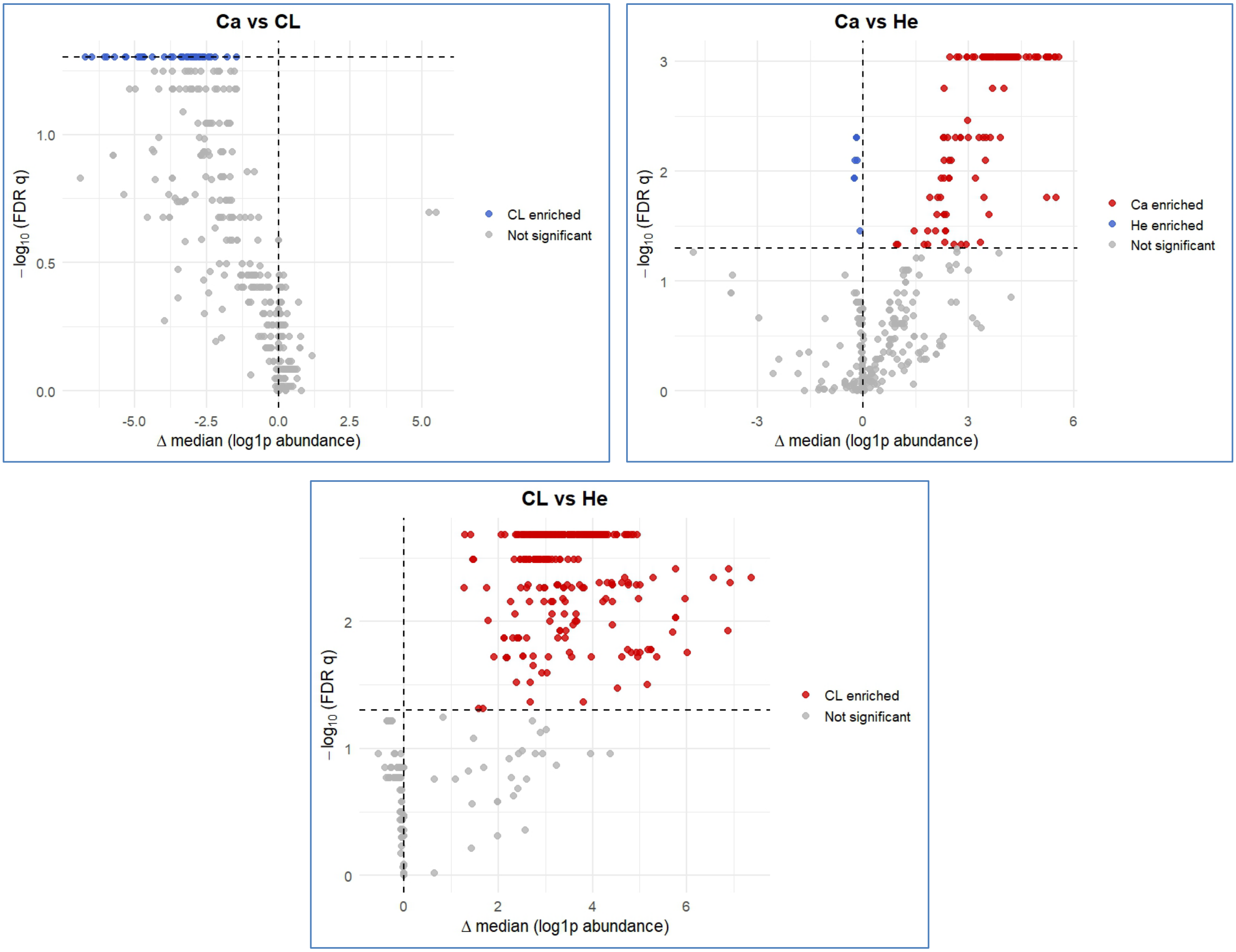

Figure S1. Volcano plots showing pairwise comparisons of predicted metabolic pathways among localities. Cancun (Ca), Comarca Lagunera (CL), Hermosillo (He). Each point represents a predicted MetaCyc pathway. The x-axis indicates the difference in median log-transformed abundance (Δ median), and the y-axis represents statistical significance (−log10 FDR-adjusted q-value). Colored points indicate significantly enriched pathways ($q < 0.05$).
